# Engaging in Creativity Broadens Attentional Scope

**DOI:** 10.3389/fpsyg.2018.01772

**Published:** 2018-09-21

**Authors:** Marta K. Wronska, Alina Kolańczyk, Bernard A. Nijstad

**Affiliations:** ^1^Faculty in Sopot, SWPS University of Social Sciences and Humanities, Sopot, Poland; ^2^Department of Human Resource Management and Organizational Behavior, University of Groningen, Groningen, Netherlands

**Keywords:** creativity, idea generation, divergent thinking, breadth of attention, self-regulation, analytic thinking, Remote Associates Test, convergent thinking

## Abstract

Previous studies have shown that creativity is enhanced by a broad attentional scope, defined as an ability to utilize peripheral stimuli and process information globally. We propose that the reverse relationship also holds, and that breadth of attention also is a consequence of engaging in a creative activity. In Study 1, participants showed increased breadth of attention in a visual scanning task after performing a divergent thinking task as opposed to an analytic thinking task. In Study 2, participants recognized peripheral stimuli displayed during the task better after performing a divergent thinking task as compared to an analytic task, whereas recognition performance of participants performing a task that involves a mix of divergent and analytic thinking (the Remote Associates Test) fell in between. Additionally, in Study 2 (but not in Study 1), breadth of attention was positively correlated with performance in a divergent thinking task, but not with performance in an analytic thinking task. Our findings suggest that the adjustment of the cognitive system to task demands manifests at a very basic, perceptual level, through changes in the breadth of visual attention. This paper contributes a new, motivational perspective on attentional breadth and discusses it as a result of adjusting cognitive processing to the task requirements, which contributes to effective self-regulation.

## Introduction

What is the temperature in the place you are currently in and what background sounds can you hear? Unless the environmental conditions are extreme, you probably did not register these peripheral, seemingly unimportant stimuli. Indeed, doing so would only be distracting and may interfere with other activities. When generating creative ideas (ideas that are both novel and useful; Amabile, 1983), however, having a broad attentional scope and noticing peripheral stimuli can be beneficial. For example, Mendelsohn and Griswold (1964) found that people who score high on creativity tests, as compared to less creative problem solvers, are better able to take advantage of peripheral cues (prompts) to solve the task at hand, and similar results were obtained in later experiments (Mendelsohn and Griswold, 1966; Mendelsohn and Lindholm, 1972; Ansburg and Hill, 2003). More recent studies also found strong support for the beneficial effect of broad attention on creative idea generation: Creativity is enhanced by meditation techniques that broaden attention (Colzato et al., 2012, 2017; see also Lebuda et al., 2016), as well as by experimental manipulations that increase attentional breadth (Friedman et al., 2003; Förster et al., 2004; Jia et al., 2009; Liu, 2016; Moraru et al., 2016). It has even been found that alcohol intake can facilitate creative proble solving, which is expected to be driven by reduced attentional control and higher sensitivity to peripheral information (Jarosz et al., 2012).

Although it is clear that breadth of attention influences creative performance, here we explore the intriguing possibility of the reverse causal relation: that engaging in creative activity can influence attentional breadth. Just as certain types of meditation or experimental manipulations enhance breadth of attention, engaging in a creative task may broaden the attentional field. This possibility is intriguing because it would suggest that the cognitive system is able to adapt to task demands at a fundamental (perceptual) level. Indeed, Vartanian (2009) suggests that successful problem solving requires the cognitive system to flexibly adjust to task requirements. Because creative tasks are ill-defined and demand exploration of problem space (e.g., Arreola and Reiter-Palmon, 2016), a broader attentional scope is beneficial and may be triggered by the particular activities for which it is needed. For example, it is possible that engaging in brainstorming activates a completely different mindset (which manifests in the attentional breadth) than engaging in planning an agenda, because planning does not require a broad search for solutions whereas brainstorming does.

The current paper reports two experiments in which we manipulated engagement in certain activities (creative idea generation or other) and measured attentional breadth in different ways. Specifically, we measured attentional breadth as a consequence of engagement in a divergent thinking task, an analytic thinking task, or (only in Study 2) in the Remote Associates Test (RAT; Mednick, 1962), a test that involves a mix of divergent and analytic thinking. Together, these studies suggest that the cognitive system adaptively responds to task demands at a very basic level of information processing (breadth of attention) and that breadth of attention is also a consequence of engaging in a creative activity. Based on these and prior findings, we propose that the attentional breadth–creativity relation is, in fact, bi-directional.

### Broad Attention Stimulates Creativity

According to Mednick’s (1962) associative theory, creativity requires finding elements that are remotely associated and combining them in a meaningful way. This theory explains why a broad scope of attention should increase creativity: It gives access to a larger pool of elements, and therefore, facilitates original combinations of these elements (Mendelsohn, 1976). Mednick proposed that people differ in the strength of their associations to certain concepts (e.g., “table”), with some people having a steep association hierarchy and others a flatter one. If one association dominates (e.g., “chair”), then the remaining, potentially creative associations are less likely to be activated, and the association hierarchy is steep; however, if various associations are similarly strong, the association hierarchy is relatively flat, which may lead to more creative outcomes. Because a broad scope of attention implies that diverse elements in the perceptual field are similarly important, broad attention should facilitate a flat association hierarchy. This idea has been proposed by Martindale (1989), who suggested that the mind can be represented as a set of interconnected nodes, similar to neural networks, which may be activated in different degrees (see also Spreading-Activation Theory of Semantic Processing; Collins and Loftus, 1975). When attention is narrow, strong activation of a single node prevents activation from spreading to other nodes in the network – in this case, a single concept (like “chair” in response to “table”) is activated strongly and adjacent nodes (like “tablecloth”) are inhibited. However, when more nodes are activated simultaneously and attention is broad, then the activation of each node is weaker, and there is no inhibiting effect on other nodes. Such situations lead to the generation of more remote, and potentially creative, associations. A similar idea has been proposed by the extensive–intensive attention theory (Kolańczyk, 1989, 1991, 2011, 2012): extensive attention relates to more sensitivity toward peripheral stimuli (rather than strong focus on central stimuli) and consequently, weak activation of a large pool of nodes in the semantic network.

Consistent with this idea, Mendelsohn (1976) found that those who are able to connect remote ideas are also those who can take advantage of seemingly irrelevant, peripheral stimuli to solve the task at hand. Furthermore, Ansburg and Hill (2003) confirmed this idea by showing that scores on the RAT (Mednick, 1962), a test which measures the ability to make remote associations, positively predict the number of word puzzles (anagrams) solved with peripheral cues (answers to the word puzzles played on the tape recorder in the background). Other evidence is also consistent with this reasoning. For example, Kasof (1997) found a positive relation between creativity of poems and sensitivity to peripheral stimuli in the environment. Experimental studies confirm that it is indeed a broad conceptual scope that increases creative performance (Isen and Daubman, 1984; Isen et al., 1987; Jarosz et al., 2012; Deuja et al., 2014; Chiu, 2015; Liu, 2016). Finally, studies on meditation suggest that attending to the surroundings in a broad and defocused manner boosts creativity: Open monitoring, compared with focused attention meditation, has been found to increase performance in creative idea generation (Colzato et al., 2012, 2017; Baas et al., 2014). Together, these findings provide converging evidence that broad attention facilitates creative performance by expanding the scope of concepts that may be combined into a potentially creative outcome.

### Does Engaging in Creativity Lead to Broader Attention?

Although it is well established that broad attention increases creativity, the idea that engaging in creative activity could alter the breadth of the attentional field has not yet been investigated. If generating creative ideas requires broadening the conceptual scope to transcend from obvious solutions to more original ones, it is also possible that attempting to produce creative output in itself will broaden the attentional field. This can be true especially when we compare it with engaging in an activity that does not require such expansion of horizons, or even asks for the opposite – focusing only on the task-relevant information to arrive at a single correct solution (cf. Ansburg and Hill, 2003; Liu, 2016).

Indirect support for this idea comes from the studies that contrasted divergent thinking tasks with the RAT (Akbari Chermahini and Hommel, 2012; Fischer and Hommel, 2012). In divergent thinking tasks, participants were asked to generate multiple creative uses of an everyday object (e.g., a brick), whereas in the RAT participants had to provide a single word that is a common associate for three words that were provided; here, only one solution was correct. Hommel (2012) argued that engaging in these tasks induces a certain control state, which either favors flexible switching between options with little “top-down” guidance (divergent thinking) or releases a strong top-down bias, which guides a person toward one specific option (solving the RAT). The first case is associated with achieving creativity through flexible and relatively effortless processing (i.e., low cognitive control and low self-control; Kolańczyk, 2012), whereas the second refers to creativity achieved through persistent and effortful processing (i.e., high cognitive control; Nijstad et al., 2010). Results have shown that engaging in divergent thinking, compared with solving the RAT, led to higher multitasking performance (Fischer and Hommel, 2012), and to a more positive mood (Akbari Chermahini and Hommel, 2012), which is associated with broad attention and global processing (Isen and Daubman, 1984; Fredrickson and Branigan, 2005; Bramesfeld and Gasper, 2008; Kuhbandner et al., 2011; Schmid et al., 2011).

### Overview of the Present Studies

Overall, these results suggest that the weak top-down control state induced by divergent thinking should be connected with defocused and broader attention (see also Martindale, 1989; Kolańczyk, 2012; Zhou et al., 2017). However, to the best of our knowledge, there is no direct evidence showing this effect. Providing a direct test of this idea is the aim of the present contribution. If indeed engaging in a divergent thinking task broadens the attentional field (as compared with engaging in analytic thinking task) this would indicate that, at a very basic perceptual level, the cognitive system can adapt to task demands.

To test the idea that engaging in creative activity leads to a broader attentional field, we performed two studies, in which we compared a divergent thinking task with an analytic thinking task (Study 1) and a divergent thinking task with both an analytic thinking task and the RAT (Study 2). We expected that performing a divergent thinking task would lead to a broader attentional field than performing an analytic thinking task, because top-down cognitive control is lower for a task that requires flexible and explorative processing (i.e., divergent thinking) than for a task that requires careful evaluation of task-related information to arrive at a single correct solution (i.e., analytic thinking). In turn, these differences in mindset and cognitive control state will translate to differences in breadth of attention.

Both studies used a between-subjects design and employed different measures of breadth of attention. Study 1 measured attentional breadth with a task specifically designed to measure extensive–intensive attention states (Roczniewska et al., 2011), with a state of extensive attention defined as broader and more sensitive to peripheral stimuli than a state of intensive attention. In the second study, we drew from the peripheral cues paradigm (Mendelsohn and Griswold, 1964) to measure breadth of attention through recognition of peripheral stimuli. We also assessed performance on each task and examined whether performance in each of the tasks correlates with our measure of breadth of attention. As discussed above, previous research suggests that breadth of attention should correlate positively with creative performance but not with analytic performance.

## Study 1

### Method

#### Participants and Design

Ninety undergraduate students participated in an experiment on the “properties of cognitive processes” in exchange for credit points. However, 14 participants were excluded from analysis due to: disrupted procedure during attention measurement (e.g., talking to the experimenter, the door being opened, noise), using a touchpad instead of a mouse, failing to understand the attentional breadth measure instruction (e.g., selecting very few stimuli, see the description of the Ellipses Test in Measures), and a computer malfunction. Data from 76 participants was analyzed (59 females and 17 males), whose age ranged from 18 to 53 years (*M* = 21.59, *SD* = 4.16). Average age did not differ between conditions, *t*(74) = 0.26, *p* = 0.798.

Participants were randomly assigned to two conditions of a between-subjects design. In the *divergent thinking* task condition (*n* = 40; 30 female, *M*_age_ = 21.48), participants performed the Unusual Uses Task with instructions developed by Silvia et al. (2008). Participants were asked to write down all original and creative uses of a brick they could think of. Participants in the *analytic thinking* task condition (*n* = 36; 29 female, *M*_age_ = 21.72) were asked to solve a task from the analytic reasoning section of the Law School Admission Test (Princeton Review, 2015; also see Kray et al., 2006). This test measures the ability to derive conclusions from a set of assumptions and asks participants to apply logic to multifaceted problems, understand how rules affect outcomes and decisions, and identify connections between concepts. The task that we employed required the participants to follow five rules (e.g., “the student must clean the kitchen first before shopping for groceries”) to determine the correct order of household chores (e.g., “grocery shopping”) performed by a student.

#### Measures

##### Breadth of attention

**FIGURE 1:**
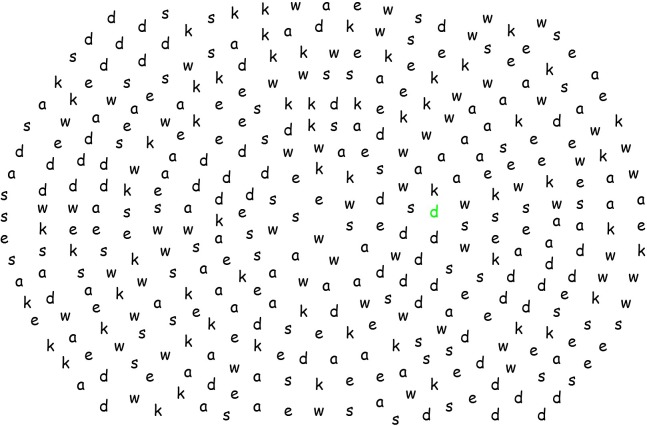
The Ellipses Test (Roczniewska et al., 2011). In this example, one letter has been selected – the *d* marked in green font.

To measure breadth of attention, we used the Ellipses Test (Roczniewska et al., 2011), which consisted of 363 letters (*a, d, e, k, s*, and *w*) arranged in the shape of ellipses on a computer screen. Ellipses made of letters varied in size, with smaller ellipses located inside bigger ellipses (see **Figure 1**). Letters were displayed in a black font on a white background. Participants had to select letters *d* with mouse clicks. After a letter had been clicked, its color turned to green to mark its selection. Some *d*s were spread out (*n* = 17) and others (*n* = 43) appeared in small clusters, which made them easier to spot with a broader attentional field. We used the distance between selections (percentages of the screen size) as indicator of attentional breadth and computed two indicators: *total distance* covered (“travelled”) by the solver while searching for *d*s and the *standard deviation* (SD) of distance between clicks.

Total distance was computed as the sum of distances between all clicks. High total distance indicates that the solver searched for *d*s globally, within a broad perceptual field; low total distance indicates that the solver searched for *d*s locally, within a narrow perceptual field. SD of distance was computed to examine the amount of variation in distances. Because most *d*s appeared in clusters and broad (but not narrow) attention should facilitate spotting such clusters, this should result in small distances within each cluster and big distances between the clusters, thus creating a high standard deviation. Participants may differ in how many letters they selected in total, so we controlled for the total number of clicked letters, as this could bias the attentional breadth indicators.

##### Control measures

It is possible that engaging in different tasks influenced participants’ mood state. Because moods affect creative performance (Baas et al., 2008), we employed two versions of a 4-item questionnaire to measure pretest and posttest mood (Wojciszke and Baryła, 2004). Participants rated statements (e.g., “I’m in a bad mood”) on a 5-point scale (1 = *disagree*, 5 = *agree*). Scale reliabilities were good (Cronbach’s α = 0.91 for version A and Cronbach’s α = 0.89 for version B). We also controlled the subjective difficulty of the task (Bujacz et al., 2014), because task difficulty may affect attentional processes (e.g., Santangelo et al., 2011; see also Santangelo and Spence, 2008). Participants indicated to what extent they found the previous task: “easy,” “undemanding,” “unproblematic” (all reverse scored), “difficult,” “complicated,” and “challenging” (Cronbach’s α = 0.85). We employed a 7-point scale ranging from 0 (*not at all*) to 6 (*very much*). Moreover, participants were asked to rate their task enjoyment (Friedman and Förster, 2002; “How much did you enjoy the task?”), using the same 7-point scale.

#### Procedure

Upon arrival, participants gave written informed consent and were randomly assigned to a divergent thinking or analytic thinking task condition. The experiment was run in Inquisit Lab4. First, participants answered four pretest mood items (Wojciszke and Baryła, 2004). Subsequently, they engaged in a divergent thinking (Silvia et al., 2008) or analytic thinking task (Princeton Review, 2015; also see Kray et al., 2006) for 1.5 min. Participants could take notes on a sheet of paper, and after 1.5 min, an audio sound signaled that they had to look at the screen again. They were asked to stop the task and were informed that they would be able to finish it later. Next, the Ellipses Test was administered to measure attentional breadth (Roczniewska et al., 2011). Participants were instructed that a number of letters would appear on the screen. Their task was to select as many letters *d* as possible with mouse clicks.

After 2 min, the test ended and the participants were instructed to finish the divergent thinking or analytic thinking task. In the divergent thinking task condition, participants continued writing down possible uses of a brick for another 1.5 min. In total (before and after the Ellipses Test), they thus performed the divergent thinking task for 3 min (see also Silvia et al., 2008). When the time was up, they had to choose their two most creative ideas and underline them. Participants in the analytic thinking task condition had 5 min to finish their task. The longer time was chosen to ensure that it was sufficient and proportional to task difficulty. However, participants were allowed to finish earlier, on condition that they had completed the task (finishing early was not allowed in the divergent thinking task condition). Participants were not informed about the time limit to avoid the confounding effect of time pressure (e.g., Hsu and Fan, 2010).

In the final part, participants rated their posttest mood with items differing from those used at the beginning (Wojciszke and Baryła, 2004). Subsequently, they evaluated the subjective difficulty and their enjoyment of the task; they indicated their gender, age, and were thanked for participation.

#### Coding Performance

##### Divergent thinking task

For the divergent thinking task, we closely followed the subjective scoring procedures developed by Silvia et al. (2008). Responses to the divergent thinking task were typed into a spreadsheet and sorted alphabetically. We engaged three coders (including the first author), all of whom were the alumni or students of an advanced university course on the psychology of creativity (including creativity diagnosis). They were trained by the first author and asked to read each response. Each coder independently scored the responses on a scale from 1 (*not at all creative*) to 5 (*highly creative*). Scoring instructions were translated from Silvia et al. (2008) by the first author and then back-translated by a professional English teacher (Polish native speaker). We obtained two indicators of creative performance: average creativity of all responses of each participant (average creativity) and an average from the two responses that the participant marked as the most creative (top 2 creativity). The interrater reliability was satifactory: intraclass correlation coefficient (ICC; two-way random model, absolute agreement) was 0.811 (*p* < 0.001) for the average creativity and 0.680 (*p* < 0.001) for top 2 creativity, which indicates good and moderate reliability, respectively (Koo and Li, 2016).

##### Analytic thinking task

The aim of the analytic thinking task was to order household chores according to rules given (Princeton Review, 2015; also see Kray et al., 2006). Two possible orders could be correctly derived from the rules. In the 0–1 indicator, participants scored one point when the entire sequence of chores was correct; otherwise, the score was 0 points. In the 0–5 indicator, one point was given for each condition that was met (e.g., if all conditions were met, the participant scored five points).

### Results

#### Control Variables

Control variables (task enjoyment and subjective difficulty, pretest and posttest mood^1^) did not differ between experimental conditions (all *t*s < 1.14; *p*s > 0.257). Mean accuracy (the number of clicked *d*s divided by the number of all clicked letters) in the Ellipses Test did not differ between conditions either, *t*(74) = 0.26, *p* = 0.794. Similarly, there were no differences between conditions in the number of all clicked letters (*t*[74] = 0.28, *p* = 0.783), number of clicked *d*s (*t*[74] = 0.35, *p* = 0.732; *M*_divergent_ = 59.58, *M*_analytic_ = 59.28), and in the number of other clicked letters (*t*[74] = 0.20, *p* = 0.845, *M*_divergent_ = 0.88, *M*_analytic_ = 0.92). This is in line with the assumptions of the method, which diagnoses attentional breadth not through the effectiveness of finding the *d*s but through the strategy of searching the perceptual field.

#### Effect of the Task on Breadth of Attention

**FIGURE 2:**
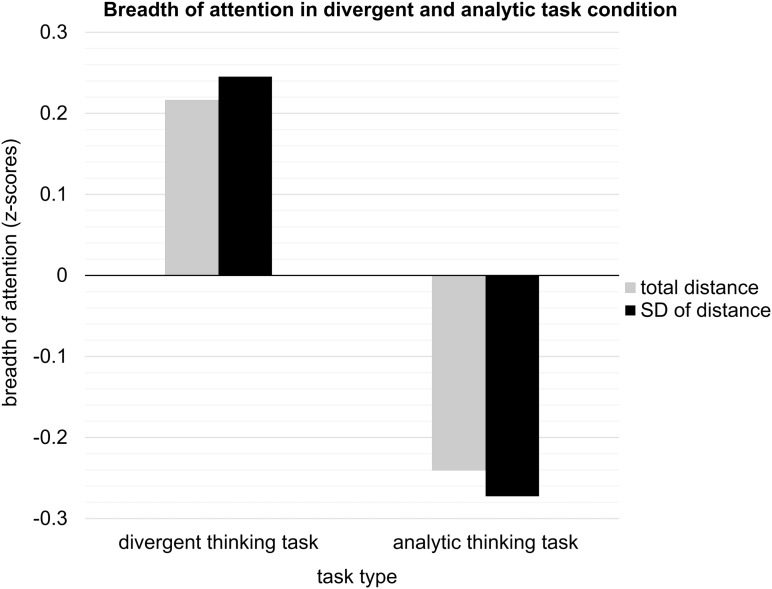
Standardized breadth of attention (total distance and SD of distance) in divergent thinking task condition and analytic thinking task condition in Study 1. Total distance (in *z*-scores) ranged from –2.41 to 2.26, and SD of distance ranged from –2.25 to 2.34.

We performed a multivariate analysis of covariance (MANCOVA) with task type (divergent vs. analytic) as independent variable, total distance and SD of distance as dependent variables, and total number of clicked letters as a covariate. We found a significant multivariate effect: participants who solved the divergent thinking task had broader attention (*M*_total_ = 962.43, *M*_SD_ = 17.56) than participants who solved the analytic thinking task (*M*_total_ = 909.81, *M*_SD_ = 16.07), *F*(2, 72) = 3.19, *p* = 0.047 (see **Figure 2**). Total number of clicked letters was a significant covariate, *F*(2, 72) = 35.57, *p* < 0.001. In a follow-up univariate analyses, the effect of task type (divergent vs. analytic) on total distance did not reach significance level when corrected for multiple comparisons, *F*(1, 73) = 3.99, *p* = 0.050 (*p* = 0.100 with Bonferroni correction), but the univariate effect of task type (divergent vs. analytic) on SD of distance was significant, *F*(1, 73) = 6.24, *p* = 0.015 (*p* = 0.030 with Bonferroni correction). Confidence intervals for both effects did not include zero, 95% CI (0.09, 102.93) for total distance and 95% CI (0.31, 2.76) for SD of distance, which suggests a significant difference for both indicators. Total number of clicked letters was a significant covariate for SD of distance, *F*(1,73) = 7.13, *p* = 0.009 (*p* = 0.018 with Bonferroni correction), but not for total distance, *F*(1,73) = 1.78, *p* = 0.186 (*p* = 0.372 with Bonferroni correction). The effect size was small to moderate (Cohen’s *d* = 0.47 for total distance; Cohen’s *d* = 0.54 for SD of distance; Cohen, 1977).

In order to verify whether mood, subjective task difficulty and enjoyment can account for the influence of the task type (divergent vs. analytic) on breadth of attention, we performed another MANCOVA. In this analysis, we additionally entered the following covariates: pretest mood, subjective task difficulty, and task enjoyment. We found that the additional covariates had no multivariate effect on breadth of attention (all *p*s > 0.13) and that the multivariate effect of the task type (divergent vs. analytic) on breadth of attention remained significant, *F*(2, 29) = 3.84, *p* = 0.026. Both effects in a univariate follow-up analyses remained at the same significance level as in the analysis without additional covariates, *F*(1, 70) = 4.02, *p* = 0.049 (*p* = 0.098 with Bonferroni correction) for total distance and *F*(1, 70) = 7.13, *p* = 0.009 (*p* = 0.018 with Bonferroni correction) for SD of distance. Thus, pretest mood, subjective task difficulty, and enjoyment cannot explain the influence of task type (divergent vs. analytic) on attentional breadth.

#### Performance

We performed a correlation analysis separately for the divergent and analytical thinking task condition to examine whether breadth of attention correlated with performance. We found that performance in the divergent thinking task, as well as in the analytic thinking task, was unrelated to total distance and SD of distance (-0.16 < *r* < 0.05; all *p*s > 0.351).

### Discussion of Study 1

Study 1 provided initial evidence that engaging in a divergent thinking task, compared with engaging in an analytic thinking task, broadens the scope of attention. We found a significant multivariate effect on attention indicators (total distance and SD of distance), both when we did and did not control for pretest mood, subjective task difficulty, and enjoyment. This suggests that these control variables cannot explain the effect of task type (divergent vs. analytic) on attentional breadth. We found a significant univariate effect on SD of distance, but the univariate effect on total distance did not reach significance. This suggests that broad attention triggered by the divergent thinking task was not so strongly visible in global search for the target letters within a broad perceptual field; instead, it was more reliably reflected in higher variation of distances obtained when the solver noticed and clicked on the *d*s that appeared in clusters. Furthermore, and somewhat surprisingly, in this study, attentional breadth was unrelated to creative performance. A possible explanation is that breadth of attention was measured in the middle of task performance. Switching attention between idea generation and the Ellipses Test potentially disrupted the flow of ideas while participants were generating creative solutions, which may have weakened the correlation between attentional breadth and creative performance.

## Study 2

To replicate the findings of Study 1 and generalize the results to other divergent and analytic thinking tasks, we performed Study 2. In this study, we wanted to avoid interrupting participants by the Ellipses Test, and therefore measured attentional breadth via the recognition of peripheral stimuli which were displayed during task performance. This method builds on the paradigm of incidental (peripheral) stimuli in creative problem solving (Mendelsohn and Griswold, 1964, 1966; Mendelsohn and Lindholm, 1972; Mendelsohn, 1976). In the studies of Mendelsohn and colleagues, participants were exposed to words played on a tape recorder while memorizing a list of other words. Next, they were asked to solve multiple anagrams. Some of the answers to the anagrams were earlier played on the tape recorder (answers to “peripheral anagrams”) and some were present on the list (answers to “central anagrams”). Those participants who achieved high scores on the creativity test also solved more peripheral anagrams (cf. Ansburg and Hill, 2003). Our study, however, used recognition of visual peripheral stimuli as a dependent variable, with the assumption that incidental recognition of peripheral cues would be better when attention is broad (vs. narrow) during task performance.

We also added a condition in which participants performed the RAT (Mednick, 1962). Interestingly, the RAT requires both divergent thinking (coming up with multiple candidates for the solution) and analytic thinking (evaluating the correctness of possible answers; Mendelsohn, 1976). Although previous research argued that solving the RAT requires more cognitive control than solving a divergent thinking task (Fischer and Hommel, 2012; Hommel, 2012), it has been found that the RAT can be solved both through an insight strategy (spontaneous activation of diverse associations) and through an analytic strategy (effortful and sequential search for close associations; see e.g., Bowden et al., 2005; Harkins, 2006). Furthermore, Topolinski and Strack (2008) found that just reading a RAT trial (three remotely associated words) triggers spreading activation in the semantic network: Participants who only read the RAT trials recognized solutions to those trials faster than unrelated, random words. However, the authors also found that intentional search for the solution *blocks* spreading activation in the semantic network: participants who intentionally searched for solutions recognized the solutions to those trials as quickly as unrelated, random words. This suggests that on the one hand, just reading RAT trials primes broad activation of the semantic network. Since such conceptual breadth translates into perceptual breadth of attention (Förster and Dannenberg, 2010), reading the RAT trial should broaden the perceptual field of attention. On the other hand, converging on a single solution should block spreading activation in the semantic network, and thus narrow the attentional field. In other words, performing the RAT may have mixed effects on breadth of attention, and therefore, we decided to explore its effects.

### Method

#### Participants and Design

One hundred thirty-eight participants were recruited through the university participant recruitment system and social networks to participate in an experiment on “solving different tasks” (107 females and 31 males). Their age ranged from 19 to 53 years (*M* = 26.79, *SD* = 8.31). Average age did not differ between conditions, *F*(2, 133) = 0.812, *p* = 0.446. To diversify our sample, in this study, apart from student participants (*n* = 97), we also recruited people who were not enrolled at university and who pursued a creative career or had a creative hobby (*n* = 38; background of three participants was not saved due to an internet connection error). Education and main activity of participants are summarized in **Table 1**. Student participants earned credit points for participation. Student and non-student participants could obtain one of seven shopping vouchers worth 50 PLN (around 12 €). Participants were seated at computers separated by screening walls in the laboratory, and were run individually or in small groups (maximally four participants).

**Table 1:** Education and main activity of participants in Study 2.

	Divergent thinking task (*n* = 47) (%)	Analytic thinking task (*n* = 45) (%)	RAT (*n* = 46) (%)	Total sample (*N* = 138) (%)
**Education**		
Primary school	0	0	2.2	0.7
High school	53.2	37.8	37.0	42.8
University level	42.5	62.2	60.8	55.1
Missing	4.3	–	–	1.4
**Main activity**		
Education	61.7	26.7	65.2	65.9
Paid work	27.7	71.1	23.9	26.1
Other	6.4	2.2	10.9	6.5
Missing	4.3	–	–	1.4

Participants were randomly assigned to three conditions of a between-subjects design: divergent thinking (*n* = 47, 38 female, *M*_age_ = 25.78), analytic thinking (*n* = 45, 33 female, *M*_age_ = 26.67), and the RAT (*n* = 46, 36 female, *M*_age_ = 28.00). As a divergent thinking task, we employed the Unusual Uses Task (Silvia et al., 2008). Participants in the analytic thinking task condition were asked to solve a task inspired by a task from the mathematical competition for pupils (Towarzystwo Upowszechniania Wiedzy i Nauk Matematycznych, 2016) and by a task used by Ansburg and Hill (2003). It required the participants to determine the order of men, from the tallest to the smallest. Participants were informed that the men have different height and different eye colors. Three premises were given, which enabled participants to derive a correct solution (e.g., “Adam is not the tallest, and Lucas does not have green eyes”). As a third condition, we used a Polish adaptation of the RAT (Sobków et al., 2016). Eight trials were included.

#### Measures

##### Breadth of attention

**FIGURE 3:**
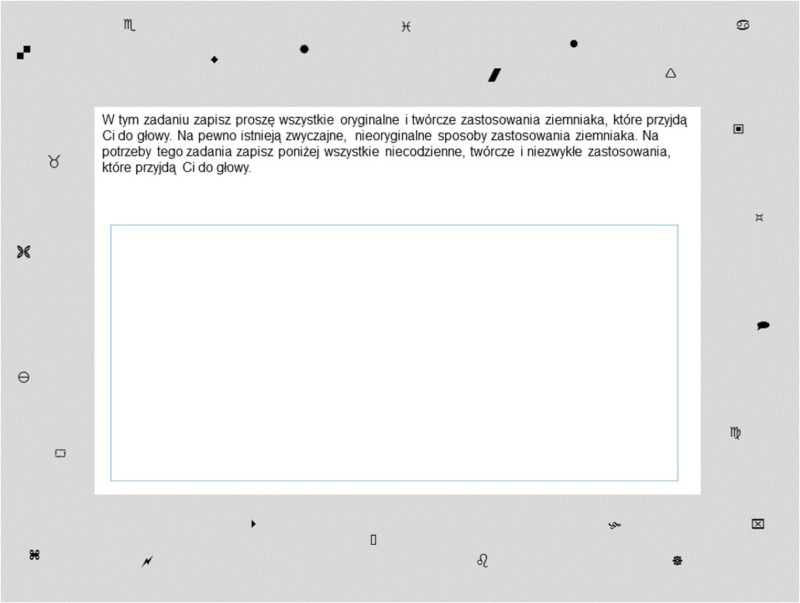
The task with instructions in the heading, response window in the middle of the screen, and symbols in the peripheries of the screen (divergent thinking task condition) in Study 2.

Participants’ task in each of the conditions was displayed in the middle of the screen on a white background, which was surrounded by a gray frame. Twenty-five peripheral stimuli, geometric shapes and symbols, were displayed on the gray frame, always in the same locations and for the duration of the whole task (see **Figure 3**; the same number of peripheral stimuli – 25 – was used in previous research, e.g., Mendelsohn and Griswold, 1964). Participants were given no information or explanation about why the symbols were there. Breadth of attention was measured by recognition of peripheral stimuli that were displayed on the screen during task performance. The recognition test started after the main task and a mood check had been completed, but participants were not informed earlier that they would perform the recognition test. It included 25 peripheral and 20 filler symbols (i.e., symbols that were not present on the screen during the task solution). Participants indicated whether a symbol was present on the screen during the task solution by pressing a number on the keyboard (1 = *definitely no*, 2 = *rather no*, 3 = *rather yes*, and 4 = *definitely yes*). We recoded these scores into 0 (*no*; score 1 or 2) and 1 (*yes*; score 3 or 4). A recognition index was computed by taking the difference between the percentage of hits (i.e., the proportion of peripheral symbols that were correctly classified as present) and percentage of false alarms (i.e., the proportion of filler symbols that were falsely classified as present). Higher recognition index scores indicate more accurate recognition, which means that attention was broader during the task solution. Possible values for this indicator vary from -100 (e.g., 0% of hits and 100% of false alarms) to 100 (e.g., 100% of hits and 0% of false alarms).

##### Control measures

We employed an Affect Grid to measure mood after the task (Russell et al., 1989). Participants were presented with a square grid divided into 9 × 9 square fields. The vertical dimension represented arousal, from sleepiness in the lower part to high arousal in the upper part, and the horizontal dimension represented valence, from unpleasant feelings on the left to pleasant feelings on the right. Participants were instructed to click on the field that reflected their feelings most accurately. In this way, we obtained two mood indicators from each participant: valence (ranging from 1 = *unpleasant* to 9 = *pleasant*) and arousal (ranging from 1 = *sleepiness* to 9 = *high arousal*). We also controlled for the subjective difficulty with an adjective scale (Cronbach’s α = 0.87 for all six items) and enjoyment of the task, using the same measures as in Study 1. Controlling for subjective difficulty is particularly important, because this may affect attention to peripheral stimuli (Santangelo et al., 2011).

#### Procedure

The study consisted of two parts: online and laboratory. In the online part, participants gave an informed consent and filled in questionnaires, results of which are not reported in this paper.^2^ Upon arrival in the lab, participants were randomly assigned to the divergent thinking, analytic thinking, or the RAT condition. The experiment was run in the Inquisit Lab5. In the first part, not reported in this paper, participants took part in another experiment, in which they also solved a divergent thinking task, analytic thinking task or the RAT. After the first part, the divergent thinking, analytic thinking task or the RAT was displayed in the middle of the screen and 25 stimuli were displayed in the peripheries. Participants remained in the same condition that they were assigned to in the first part (i.e., they solved a different task of the same type for the second time).

In the divergent thinking task condition, people generated creative uses of a potato (Silvia et al., 2008) and entered their ideas in the field located in the middle of the screen. The time limit was not mentioned, and the task automatically terminated after 180 s. In the analytic thinking task condition, participants ordered four men from the smallest to the tallest, based on the premises given (Ansburg and Hill, 2003; Towarzystwo Upowszechniania Wiedzy i Nauk Matematycznych, 2016). Their task was to write the names of men in the correct order in the field located in the middle of the screen. The time limit set for this task – 360 s – was not mentioned. The longer time was chosen to ensure that it was sufficient and proportional to the difficulty. However, participants were allowed to finish earlier, on condition that they completed the task (which was not allowed in the divergent thinking task condition). In the RAT condition, participants solved eight RAT trials (Sobków et al., 2016). One trial consisted of three words and a response field displayed on a single screen. The time limit was not mentioned, and each trial automatically terminated after 30 s (Sobków et al., 2016). However, participants were allowed to finish earlier, on condition that they completed the trial. We intended to provide participants in each of the conditions with a similar amount of time to solve the task. Solving all eight RAT trials could take a maximum of 240 s, which was similar to the solution time in other conditions.

Next, all participants performed a mood check (Affect Grid; Russell et al., 1989), and proceeded with the recognition test (“Was this stimulus present on the screen?”). One stimulus at a time was displayed on the screen, and participants responded to all 25 peripheral and 20 filler stimuli in random order. In the end, participants indicated their gender and age, and were thanked for participation.

#### Coding Performance

##### Divergent thinking task

We trained three independent coders to score three classic indicators of creativity: fluency, flexibility, and originality (Guilford, 1950, 1967). Participants’ responses were typed into a spreadsheet and sorted alphabetically. To obtain fluency measure, the coders counted all generated ideas. To score flexibility, the coders classified each idea into one of 15 categories predefined by the first author and verified with other coders before scoring (e.g., “using potato as a container: making some kind of a container from a potato, where other objects can be stored”). Flexibility of a participant was the number of non-redundant categories in which we could classify the responses. Originality of an idea was rated on a scale from 1 (*not original at all*) to 5 (*very original*), with an original idea defined as “an idea that is infrequent, novel, and original.” Therefore, coders were asked to bear in mind both the objective frequency of a specific idea in a sample, as well as subjective novelty and originality. Originality of a participant was the average originality of all participant’s ideas. A similar coding procedure was employed by De Dreu et al. (2008). The interrater reliability was high: ICC (two-way random model, absolute agreement) for fluency = 0.999, *p* < 0.001, for flexibility ICC = 0.940, *p* < 0.001, and for originality ICC = 0.845, *p* < 0.001 (Koo and Li, 2016). We used the average scores across raters as indicators of divergent thinking performance.

##### Analytic thinking task

The aim of the analytic task was to order men from the lowest to the tallest (Ansburg and Hill, 2003; Towarzystwo Upowszechniania Wiedzy i Nauk Matematycznych, 2016). Four men were listed in the task. Establishing the correct order required deriving three correct pairings (e.g., Rafael→Adam, Adam→Michael, Michael→Lucas). One point was given for each correct pairing. Therefore, participants could score between 0 and 3 points for the analytic thinking task.

##### The RAT

Participants scored 1 point for each correctly solved trial (Sobków et al., 2016). Therefore, participants could score between 0 and 8 points in the RAT condition.

### Results

#### Control Variables and Solution Time

We performed separate analyses of variance (ANOVAs) to examine the effects of our manipulation on task enjoyment, subjective difficulty, valence, and arousal, and found no effects (all *p*s > 0.128). On average, participants spent 144 s on solving analytic thinking task (*SD* = 57 s) and 144 s on solving the RAT (*SD* = 76 s). The solution time of divergent thinking task was fixed and was 180 s.

#### Effect of the Task on Breadth of Attention

**FIGURE 4:**
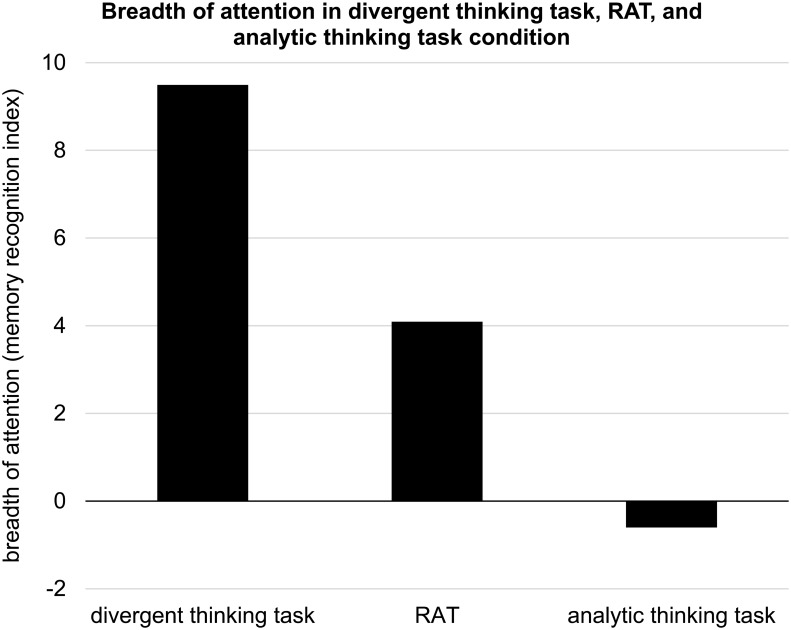
Breadth of attention (memory recognition index: percentage of hits minus percentage of false alarms) in divergent thinking task condition, the RAT, and analytic thinking task condition in Study 2.

We performed a one-way ANOVA with task type (divergent thinking vs. analytic thinking vs. RAT) as independent variable and the recognition index as dependent variable. We found a significant difference in the memory recognition index among the three conditions, *F*(2,135) = 4.25, *p* = 0.016 (see **Figure 4**). A follow-up simple effects analysis revealed that recognition in the divergent thinking condition (*M* = 9.49, *SD* = 17.03) was significantly higher than in the analytic thinking condition (*M* = -0.60, *SD* = 11.95, *p* = 0.013 with Bonferroni correction). Confidence interval for this comparison did not include zero, 95% CI (1.69, 18.49), and the effect size was moderate (Cohen’s *d* = 0.69). Recognition in the RAT condition (*M* = 4.09, *SD* = 19.79) did not differ significantly from the other conditions (*p*s > 0.357). Confidence intervals for comparisons between RAT and other conditions included zero, 95% CI (-2.95, 13.75) with divergent thinking task and 95% CI (-3.75, 13.13) with analytic thinking task.

In order to verify whether time on task, mood, subjective task difficulty, and enjoyment can account for the influence of the condition (divergent thinking vs. analytic thinking vs. RAT) on breadth of attention, we performed analysis of covariance with condition as independent variable, recognition index as a dependent variable and solution time, valence, arousal, subjective task difficulty, and enjoyment as covariates. All covariates apart from subjective task difficulty were not significant (*p*s > 0.101). However, subjective task difficulty was a significant covariate, *F*(1,129) = 5.44, *p* = 0.021, but the effect of manipulation (divergent thinking vs. analytic thinking vs. RAT) on recognition index remained significant after controlling for covariates, *F*(2, 129) = 5.48, *p* = 0.005.

#### Performance

We performed a correlation analysis separately for each condition to test whether recognition (the index of attentional breadth) is related to performance in each of the conditions. Performance was related to recognition in the divergent thinking task condition (*r*_flexibility_ = 0.34, *p* = 0.019; *r*_originality_ = 0.29, *p* = 0.047, *n* = 47) and the RAT condition (*r* = 0.44, *p* = 0.002, *n* = 46), but not in the analytic thinking task condition (*r* = 0.14, *p* = 0.352, *n* = 45).

### Discussion of Study 2

Using a different measure of attentional breadth, Study 2 conceptually replicated the findings of Study 1 and strengthened the evidence that engaging in divergent thinking tasks, compared with engaging in analytic thinking tasks, broadens the scope of attention. Interestingly, attentional breadth triggered by the RAT did not differ significantly from attentional breadth triggered by other tasks. A reason for this may be that the RAT can be solved with different strategies that employ more divergent or analytic thinking (Jung-Beeman et al., 2004; Bowden et al., 2005); therefore, the effects of engagement in RAT may vary depending on the method of solution. Furthermore, while reading the RAT triads should broaden attentional field, looking for a single solution is more likely to narrow the attentional breadth (Topolinski and Strack, 2008), and this make the effects of RAT more similar to the effects of divergent thinking and analytic thinking task, respectively. This also implies that the differences in top-down control state caused by divergent thinking and the RAT may show up in contexts that favor strong but not weak top-down control. For example, these effects may be more pronounced when attention is measured with tasks that favor narrow attentional breadth (see Fischer and Hommel, 2012).

In this study, we found the expected positive relationship between breadth of attention and performance in divergent thinking task. In contrast to Study 1, task performance was not interrupted in this study, and this seems a plausible reason why the correlation was stronger than in Study 1. Similarly to previous findings, analytic thinking performance did not correlate with attentional breadth (Ansburg and Hill, 2003; Liu, 2016). However, we also found a positive relationship between attentional breadth and the RAT performance, which is consistent with the idea that a weaker top-down control state facilitates finding remote associates (cf. Martindale, 1989; Kolańczyk, 2011; Kenett et al., 2014).

## General Discussion

The ability to process peripheral stimuli together with the ability to broaden the attentional field has been suggested to characterize creative problem solvers (Mendelsohn and Griswold, 1964, 1966; Mendelsohn and Lindholm, 1972; Kasof, 1997; Ansburg and Hill, 2003; Zmigrod et al., 2015). What is more, evidence from experimental research has shown that attentional breadth has a causal effect on creativity (Friedman et al., 2003; Förster et al., 2004; Jia et al., 2009; Colzato et al., 2012, 2017; Liu, 2016; Moraru et al., 2016). However, attentional breadth has not been examined as a *result* of engaging in a creative activity. The present research shows that engaging in creative idea generation indeed broadens the scope of attention compared with engaging in analytic thinking and that this broadened attention relates to higher creative performance. These results suggest that the adjustment of the cognitive system to task demands manifests at a fundamental, perceptual level, through changes in breadth of visual attention. Below, we interpret these results in terms of self-regulation, discuss the limitations of our studies, and suggest questions for further research.

### Attentional Breadth as a Self-Regulation Mechanism

Showing the reversed causal relationship between creativity and attentional breadth provides a new perspective, in which attentional breadth has a motivational basis. In this view, attentional breadth is a result of adjusting cognitive processing to task requirements, which ensures effective self-regulation (cf. Bargh et al., 2001). During task engagement, people represent task requirements as their goals, and these goals regulate cognitive processing (e.g., Locke and Latham, 2002; Ferguson et al., 2008). We extend this line of research by showing that attentional breadth results from specific task requirements and may play a self-regulatory role.

This perspective is consistent with several theoretical approaches. For example, the extensive–intensive attention theory (Kolańczyk, 2011, 2012) suggests that ambiguous and ill-defined goals (as in creative idea generation tasks) trigger broad attention, while specific goals (as in analytic thinking tasks) narrow the field of attention. When a goal is ill-defined, broad attention enables exploration and flexibility, which in turn facilitates goal attainment (cf. Johnson et al., 2006). The broaden-and-built theory of positive emotions also points to a similar function of broad attention (Fredrickson and Branigan, 2005). It postulates that attentional breadth results from emotions, with positive emotions broadening the scope of attention and providing room for exploration and novel behaviors. This self-regulatory role of attentional breadth has also been found in the present research: Creative idea generation led to broader attention than analytic thinking, and broad attention was related to increased creative performance. Therefore, attentional breadth seems to align with task requirements, which may support effective self-regulation and goal attainment.

This line of reasoning is also compatible with construal level theory (Trope and Liberman, 2010), which posits that people represent objects at lower, concrete levels or at higher, abstract levels. The level of representation depends on psychological (temporal or physical) distance between the self and represented objects: the greater the distance, the more abstract and broad object representations. Therefore, construal level adjusts to psychological distance, similar to how attention adjusts to task demands. Indeed, studies have shown that greater temporal, physical, or social distance facilitates global (vs. local) processing, and thus broadens the attentional field (Liberman and Förster, 2009). Similar to broad attention increasing creative performance, temporal (Förster et al., 2004) and physical distance (Jia et al., 2009) also increase creative performance. Therefore, our results are consistent with construal level theory findings. Engaging in creative idea generation, compared with engaging in analytical thinking, is likely to elicit simultaneously higher level construals and a broader attentional field; however, the interdependence of these effects is yet to be examined.

### Limitations

Results of our experiments have to be interpreted in the light of some limitations. A first limitation is that the two studies differed on various aspects, including different divergent thinking and analytic thinking tasks (and the inclusion of the RAT only in Study 2), and different measures of attentional breadth. In Study 1, task performance was interrupted and breadth of attention was measured with a separate task, but in Study 2, participants encoded the symbols during the task performance and later reported their recognition of symbols. Therefore, attention was measured without interrupting task performance in Study 2 and participants were not even aware that the recognition of symbols would later be measured. Although findings were consistent in that the divergent thinking task in both studies led to broader attention than the analytical task, one finding was clearly different: breadth of attention correlated with divergent thinking performance in Study 2 but not in Study 1. It is likely that this correlation was not obtained in Study 1 because the measurement of attentional breadth interfered with performance on the divergent thinking task.

Second, we established our effects of type of task on breadth of attention using between-participants designs. We cannot exclude that *a priori* differences between conditions existed in breadth of attention, although such differences should be eliminated by random assignment of participants to conditions. Nonetheless, within-participants designs would offer the opportunity to observe changes in breadth of attention as a consequence of performing a certain task, which would offer strong evidence for the effects of task performance on breadth of attention. One difficulty, however, with such a design is that breadth of attention had to be measured twice, and preferably with similar tasks, which may be problematic because of learning effects (e.g., peripheral stimuli might be intentionally memorized if the recognition test was anticipated).

Third, and related, although we found that performing a divergent thinking task led to higher breadth of attention than performing an analytical task, we cannot conclude whether the divergent task increased breadth of attention or the analytical task lowered it. Again, a within-participants design may solve the issue. Alternatively, some control condition could be used, although it is not clear *a priori* which tasks would have no effect on breadth of attention and could function as a neutral control condition.

### Future Directions

Besides addressing these limitations, we also see other opportunities for future research. An interesting issue relates to different manifestations of cognitive adjustment to task demands. One line of research has linked creativity to the tendency toward global vs. local processing (i.e., whether people perceive an object as a whole or whether they attend to the details of the object; Navon, 1977; Förster and Dannenberg, 2010). Our findings indicate that engaging in creativity can increase breadth of attention, which may relate to more global processing (cf. Hommel, Akbari Chermahini, van den Wildenberg, and Colzato, unpublished manuscript). This was visible, for example, in the identification of clusters of target letters in Study 1, and “jumping” among these clusters, rather than engaging in local and sequential search for single target letters. However, other research has linked creativity to breadth of attention through the functioning of an “attentional filter” (Mendelsohn and Griswold, 1964; Mendelsohn and Lindholm, 1972; Zabelina et al., 2015, 2016). This work proposes that creativity benefits from a “leaky” attentional filter, which allows peripheral stimuli to enter the field of attention. Thus, engaging in a creative task may also lead to increased sensitivity to peripheral cues and a more “leaky” attentional filter, which is consistent with our findings regarding recognition of peripheral stimuli in Study 2. Perhaps *the same* adaptation of attentional breadth to the ongoing situational demands can manifest in *different* ways, and therefore, can be captured with different methods. This is in line with self-regulatory role of attention, and future research can examine this issue more closely.

Future research could also clarify the role of the orienting mechanism of attention – how attention aligns with an internal (e.g., memory structure) or an external sensory (e.g., object from the surroundings) stimulus (Posner, 1980). This mechanism consists of overt and covert orienting. Overt orienting can be observed through head and eye movements, whereas covert orienting occurs when the object of attention changes without eye or head movements. Broad attention can be achieved both through overt (exploring the environment with multiple fixations while thinking about the task solution) as well as covert orienting (enhanced peripheral vision through more global processing while still fixating on the task). Both mechanisms may be responsible for our results, and future work could examine this.

Importantly, Santangelo et al., 2011 (see Santangelo and Spence, 2008 for a review) found that covert orienting toward peripheral cues depends on how (objectively) difficult the main task is in terms of perceptual load (amount of information to be attended to). Even though we controlled for subjective task difficulty, it is likely that perceptual load in each of the tasks in Study 2 was different. For example, the divergent thinking task consisted of a general instruction (“write down all original and creative uses of a brick”) but had no further restrictions (low perceptual load), whereas the analytic thinking task consisted of a task instruction and a set of restrictions which had to be respected in order to reach the solution (high perceptual load). It is therefore possible that perceptual load was responsible for the effect in Study 2. However, in Study 1, attentional breadth was measured independently from the main task – perceptual load during the Ellipses Test was identical across conditions – which is inconsistent with this alternative explanation. Nevertheless, future studies could provide a more nuanced perspective on attentional breadth triggered by divergent and analytical thinking tasks, through manipulations of perceptual load.

Finally, it would be interesting to further investigate the relationship between attentional breadth and solving the RAT (Mednick, 1962). Although we did not detect a difference in the attentional breadth triggered by the RAT vs. other tasks, we did find a positive relationship between breadth of attention and the RAT performance. A possible explanation is that the RAT may involve characteristics of both divergent thinking (e.g., employing various strategies in the search for the solution) and analytic thinking (e.g., arriving at a single correct solution through careful examination of existing options). In contrast to analytic thinking tasks, the pathway to the solution in the RAT is not straightforward and the most obvious associations are often not correct, which requires the solver to look for solutions in multiple directions. Therefore, the RAT may benefit from broad attention, especially when the correct solution is remotely related to all of the three provided words; at the same time, it may trigger narrow attention, because the task instruction emphasizes the goal of finding the single correct solution (cf. Topolinski and Strack, 2008). This explanation is partly supported by Harkins (2006), who showed that inducing greater effort facilitates performance in easy RAT items and inhibits performance in difficult RAT items. The author found that the activation of close associates – narrow attention – was responsible for worse performance on difficult items under increased effort. Further work could examine whether the positive relationship between attentional breadth and the RAT performance holds only for the difficult RAT items or whether it depends on the solution strategy (insightful vs. analytic; Bowden et al., 2005). Additionally, it would be interesting to examine how only reading RAT trials vs. reading and searching for the solutions affects attentional breadth.

### Conclusion

The present research showed that engaging in creative idea generation, as compared with engaging in analytic thinking, broadens the scope of attention. Interestingly, we found that broadened attention also relates to higher performance in creative tasks. Our findings converge with the control-state approach to creativity (Hommel, 2012), in which engaging in creativity triggers stronger or weaker “top-down” guidance, and spills over into how subsequent tasks are performed, depending on whether the goal is to produce multiple different ideas or to arrive at a single correct answer. The present findings shed light on attentional breadth as a self-regulation mechanism: we show that activating a goal embedded in a task leads not only to adjustment of attentional breadth, but that this adjustment may also support task performance. As such, this work indicates that the cognitive system is highly adaptable to task demands and that such adaptation can be observed at the basic, perceptual level, through changes in breadth of visual attention.

## Data Availability

The raw data supporting the conclusions of this manuscript will be made available by the authors, without undue reservation, to any qualified researcher.

## Ethics Statement

These studies were carried out in accordance with the recommendations of the ethical standards, Ethical Research Committee of SWPS University of Social Sciences and Humanities, Faculty in Sopot, Poland. The protocol was approved by the Ethical Research Committee of SWPS University of Social Sciences and Humanities, Faculty in Sopot, Poland, project number WKE/S 14/V/6. All subjects gave informed consent in a written (Study 1) or online (Study 2) form in accordance with the Declaration of Helsinki.

## Author Contributions

MW and AK developed the theoretical conception and the study design. MW performed the data collection and analyses. MW, AK, and BN interpreted the results. MW drafted the manuscript. BN and AK revised the manuscript and contributed to the final version.

## Conflict of Interest Statement

The authors declare that the research was conducted in the absence of any commercial or financial relationships that could be construed as a potential conflict of interest.

## Footnotes

1 Additionally, we tested whether the divergent thinking task triggered a more positive mood and the analytic task triggered a more negative mood, as reported by Akbari Chermahini and Hommel (2012). We performed a two-way repeated measures ANOVA with task type as a between-subjects factor (independent variable) and mood as a within-subjects factor (dependent variable). We found a significant interaction between task type and mood *F*(1,74) = 11.77, *p* = 0.001. A follow-up simple effects analysis revealed that solving a divergent thinking task induced a more positive mood (*M*_pretest_ = 3.79. *M*_posttest_ = 3.97, *p* = 0.044, Cohen’s *d* = 0.39), whereas solving an analytic thinking task elicited a more negative one (*M*_pretest_ = 4.04, *M*_posttest_ = 3.79, *p* = 0.007, Cohen’s *d* = 0.66). This is in line with findings of Akbari Chermahini and Hommel (2012).

2 Details on these measures may be obtained from the first author.
